# Towards explainable community finding

**DOI:** 10.1007/s41109-022-00515-6

**Published:** 2022-12-08

**Authors:** Sophie Sadler, Derek Greene, Daniel Archambault

**Affiliations:** 1 Swansea University, Swansea, UK; 2 School of Computer Science, University College Dublin, Dublin, Ireland

**Keywords:** Network analysis, Graph mining, Community detection, Explainability

## Abstract

The detection of communities of nodes is an important task in understanding the structure of networks. Multiple approaches have been developed to tackle this problem, many of which are in common usage in real-world applications, such as in public health networks. However, clear insight into the reasoning behind the community labels produced by these algorithms is rarely provided. Drawing inspiration from the machine learning literature, we aim to provide post-hoc explanations for the outputs of these algorithms using interpretable features of the network. In this paper, we propose a model-agnostic methodology that identifies a set of informative features to help explain the output of a community finding algorithm. We apply it to three well-known algorithms, though the methodology is designed to generalise to new approaches. As well as identifying important features for a post-hoc explanation system, we report on the common features found made by the different algorithms and the differences between the approaches.

**Supplementary Information:**

The online version contains supplementary material available at 10.1007/s41109-022-00515-6.

## Introduction

Explainability is a growing area of study in machine learning, due to the “black-box” nature of many algorithms in this field (Adadi and Berrada 2018). Models can have millions of parameters, which rely on long training processes for optimal tuning. This often results in a lack of understanding as to why a model returns the outputs it does, and there is a growing concern among experts that this can lead to hidden biases in the trained models. Even when such flaws are present in the model’s reasoning, these can remain undetected due to its good performance on a specific set of data. To combat this problem, machine learning experts have been developing techniques that provide explanations for the outputs produced by a trained model (Wachter et al. 2017).

Most Explainable AI (XAI) techniques have focused on algorithms which are typically applied to tabular or image data. However, black-box algorithms, which provide little explanation for their outputs, also exist in tasks outside of the traditional realm of machine learning. Network analysis is another field which relies on complex, stochastic algorithms to solve its well-known problems. One such problem is that of community finding, also known as community detection. In network analysis, relational data is represented by a graph structure, where data points known as nodes are connected by edges. Communities in this context are loosely defined as sets of densely-connected nodes in the graph, where connections between the identified sets are more sparse. These sets may overlap, although more commonly algorithms partition the nodes into disjoint communities, which is our focus here. In previous work, the definition of a community has sometimes varied slightly depending on the domain context. Nevertheless, a range of well-known algorithms have been developed to try and solve the problem of identifying community structure in networks (Fortunato 2010). Although these algorithms aim to optimize a quality function, usually through a heuristic, this optimization process can be very complex, leaving even experts with little intuitive understanding of the outputs. As with machine learning algorithms, little to no explanation for these outputs is provided.

One common factor which prevents a machine learning model’s performance being reasonably understood is the presence of many (sometimes hundreds or thousands) of input features. Therefore, some of the popular techniques in explainability have focused on the idea of feature importance—i.e., generating an explanation for a previously-constructed model in the form of a set of important features and their relative importance (Guidotti et al. 2018; Saarela and Jauhiainen 2021). In general, such explanations ideally present the user with features that are readily interpretable. Here an interpretable feature can be considered to be one which carries meaning to a human domain expert, such as a patient’s temperature to a doctor. This is in contrast to an uninterpretable feature, such as an individual pixel in an image. Interpretable features can be used to improve understanding in many ways, including but not limited to incorporating them as the inputs to a simpler, surrogate model which mimics the performance of the one to be explained (Keane and Kenny 2019). In a community finding context, some nodes consistently participate in a single community (a core node of that community) across several runs of the algorithm, whereas others may oscillate between two or more communities. At the moment, what distinguishes these two node types remains opaque to expert users, without network features to explain it. However, it may be that if one were to know that a node is of high betweenness centrality, one could infer that this node would likely oscillate, whereas a node with high clustering coefficient may not. This said, whether the current set of social network analysis metrics can be reliably used across algorithms to explain such phenomena, including the question of whether such a set even exists, remains unknown.

Outside the direct application of this work to social networks, explainable network analysis, and in particular, explainable community finding, would bring benefits for helping public health network interventions. In this area, social network analysis is used to understand phenomena with social components and accelerate behaviour change (e.g. alcohol misuse). Interventions are designed by using social network analysis metrics and community finding algorithms (Valente 2012; Park et al. 2020; Hunter et al. 2015, 2019; Valente et al. 2013, 2015; Gesell et al. 2013) as well as developing understanding of how these phenomena spread through a community, known as social contagion (Valente and Yon 2020; Brown et al. 2017). Therefore, as researchers in this field are well versed in social network metrics, permitting more explainable community finding results would bring benefits to studies in these areas.

The incorporation of interpretable features in a post-hoc explanation may be one promising approach for improving our understanding of community finding algorithms. In particular, we focus on interpretable features which can be understood by end-users who have social network analysis expertise and wish to apply community detection techniques to applied problems such as those in public health. Thus, our contributions are:

- A novel methodology for identifying those interpretable features which provide most insight into the outputs from stochastic community finding algorithms, from an initial longlist of candidate features.
- An application of this methodology to three well-known community finding algorithms in two experiments, and thus a list of interpretable features which relate to their performance.
- A discussion of the insight gained into these algorithms from the results.

In our experiments, we find that the same features are identified across three algorithms, indicating common underlying optimisation among these algorithms, as well as a basis for believing that these features are relevant for explainable community finding as a whole. At the single node level, these features were: clustering coefficient; triangle participation; eigenvector centrality; and expansion. At the node-pair level, these features were: the Jaccard coefficient; the cosine similarity; and to a lesser degree, the maximum betweenness centrality of an edge along the shortest path between the two nodes. All of these features are defined in “Problem formulation” section.

As well as the insight gained here by the identification of the relevant features, we also envision that our proposed approach could be incorporated into future work which generates detailed explanations for the communities found in specific graphs.

## Background and related work

### Explainable AI

Recently there has been an extensive interest in explaining the outputs of “black-box” AI algorithms, frequently referred to as Explainable AI (XAI) (Adadi and Berrada 2018). One strand of this work has prioritised “model transparency” (Rudin 2019), where the core idea is that a model is transparent only if we can understand its inner workings. However, for certain types of data or for more complex algorithms, such an approach might not be feasible or effective. As an alternative, “post-hoc explanations” have become popular in the field of XAI, which are more concerned with why an algorithm produced a given output, and usually involve providing some kind of rationale or evidence for that output (Lipton 2018; Bach et al. 2015; Fong and Vedaldi 2017; Sundararajan et al. 2017). Work by Lundberg and Lee (2017) provided a unified framework for interpreting predictions by assigning each input feature an importance value for a particular input. This approach is based on the early work by Shapley (2016). Another of the most well-known post-hoc approaches is local interpretable model-agnostic explanations (LIME), proposed by Ribeiro et al. (2016), which tries to understand a previously-built model by perturbing the input data to see how the resulting predictions change. The output is a set of local feature importances which explain the classification of a specific observation. The authors also introduced SP-LIME (submodular pick LIME), which differs in that it provides global explanations for the model as a whole, rather than for individual observations. Both approaches are model-agnostic in the sense that they can be applied in conjunction with any classifier and do not require inspecting the internal workings of that classifier.

Other work in post-hoc explanation by Keane and Kenny (2019) examined the use of a *twin-systems* strategy, where a complicated neural network model is mapped to a simpler, more interpretable “twin” model. This allowed the authors to understand the outputs of the former “black-box” model by using the latter “white-box” model.

Despite the extensive attention paid to XAI in recent years, the majority of this work has focused on either image or tabular data. In particular, little attention has been paid to tasks involving network data. Some initial work has begun to incorporate explainability into graph neural networks (GNNs) (Ying et al. 2019; Yuan et al. 2020), but network analysis tasks such as community detection remain unexplored, though there is some work on a similar problem in clustering algorithms (Morichetta et al. 2019; Loyola-Gonzalez et al. 2020). In this paper, our focus is specifically on community finding techniques for network data, rather than classification. We aim to identify sets of useful features which can allow us to explain the outputs of these algorithms in a post-hoc manner.

### Community finding

As discussed, there has been little work on explainability or interpretability for community finding algorithms and network analysis in general, to the best of our knowledge. However, existing work on comparing the performance of several algorithms on benchmark graphs has guided our choice of algorithms and data for the experimental evaluation of our proposed features. Lancichinetti et al. (2008) propose the LFR benchmark graph dataset generator, which creates graphs with ground truth community labels on each of the nodes. They assume that both the node degrees and the community sizes follow power law distributions, and define a mixing parameter, $\mu$, which introduces noise to the communities relative to its value. For low values of $\mu$, the communities remain well separated and thus easy to detect, but as the mixing parameter increases, communities become harder to identify. In a subsequent paper (Lancichinetti and Fortunato 2009), the performance of several well-known community finding algorithms is then compared on this benchmark data. Lee and Archambault (2016) find that humans behave in a similar way to Lancichinetti et al. when observing their own social network, confirming that the Infomap, Louvain and Girvan-Newman algorithms were the best-performing. This led to our decision to include Infomap (Rosvall and Bergstrom 2008) and the Louvain algorithm (Blondel 2008) in our experimental evaluation.

Previous work in computational social science has also compared the performance of community finding algorithms on other datasets, including real data (Dao et al. 2020; Ghasemian et al. 2019; Peel et al. 2017). For this study, we choose to focus on the LFR data as it allows us to generate much larger datasets and to vary the mixing parameter to observe its effect on the results. The study by Bothorel et al. (2020) proposes a methodology to describe results of the algorithm to non-experts, however this differs from ours in that their aim is to assist in making a choice of algorithm for a particular problem, not to specifically explain the algorithm’s results.

In addition to the LFR data and the community detection algorithms needed for our experiments, we also rely on the notion of the a node’s ease of clustering. Nodes which are easy to cluster are those which are consistently assigned to the same community, while a node which the algorithm finds it hard to cluster will oscillate between two or more communities across successive runs. Existing literature in this vein originates in papers unrelated to networks and community finding, but focused on more general clustering algorithms, e.g. *k*-means clustering (von Luxburg 2010; Ben-David et al. 2007). However, our proposed work differs from theirs as we centre our definition of a node’s ease of clustering on its entropy in a coassociation matrix. Entries in the matrix describe how frequently two nodes are clustered into the same community. The concept of a coassociation matrix describing the relationship between pairs of nodes was derived from work proposed by Strehl (2002). In a similar approach, the authors of the LFR benchmark explore the use of consensus clustering to determine community structure over successive runs of the algorithm (Lancichinetti and Fortunato 2012a). Other works addressing the consistency of community finding algorithms (Chakraborty et al. 2013; Francisco and Oliveira 2011) are not directly relevant to our node feature experiments described in this paper, but may have relevance to the future work we propose in identifying community features.

## Problem formulation

Due to their widespread adoption and suitability for our proposed methodology, in our experiments we focus on stochastic algorithms, where the community structure can change between successive runs, and on algorithms which find node partitions (i.e. each node belongs to exactly one community). Extending our approach to algorithms which generate overlapping communities will require additional steps, so we reserve this for future work. As the intention is to identify features which contribute intuitive understanding, our emphasis is on selecting features which are simple and easily understood to end-users, though specifically those with social network analysis expertise. We propose a model-agnostic methodology which can be adapted to any stochastic algorithm of interest, however we test it here on three in particular.

We distinguish between two “levels” of graph feature, allowing for understanding of the nodes’ community membership from two different perspectives. The first of these is at the *node-level*. Features at this level are calculated for individual nodes of the graph, with the aim to understand the community membership of that specific node. To motivate this problem in a social network context, suppose a node is occasionally classified as belonging to a community with extremist views on certain runs of a community finding algorithm. Understanding why this node has this varying classification would be important as this classification is not certain and could have important repercussions for the individual. The second is at the *node-pair-level*, where features are calculated for pairs of nodes. The aim is to understand why two nodes belong to either the same or different communities. In a social network context, if two nodes belong a community that holds an extremist view, it is important to understand why they have been placed into this community. Similarly, if one node belongs to this community and another does not, it is important to understand why the nodes have been separated.

In this work we use a large number of synthetically-generated graphs to verify our approach. We employ the use of synthetic data to ensure the results are not a consequence of the characteristics of a single network (as real data is sparsely available) and to allow us to vary parameters of the network structure consistently to observe how these parameters affect the results. However, with the aim to apply these results to real data in the future, we use a synthetic generation process which can closely mimic the observed structure of real-world networks. Specifically, the synthetic graphs were generated using the LFR benchmark algorithm (Lancichinetti et al. 2008). An additional benefit of this approach is that existing work has already evaluated the performance of community finding algorithms on LFR graphs. We use several values of the LFR mixing parameter $\mu$ in order to ascertain whether the separation of the communities affects the identified features.

Our approach is to identify a longlist of features at both the node-level and the node-pair-level. We then use these features as the input data for a classification task, and extract the most informative features using permutation importance (Algorithm 1) for our trained model. Since some of the features in our longlist depend on nodes’ community labels, we calculate these from many runs of the community finding algorithm using a mean average. If the feature does not depend on community label, it can be directly calculated once for each node or pair of nodes. To inform our choice of features, we consult the survey by Chakraborty et al. (2017) and select features which are widely adopted, state-of-the-art metrics for community evaluation such that they can be easily recognised and interpreted by experts on network analysis. The features selected for our experiments are described below.
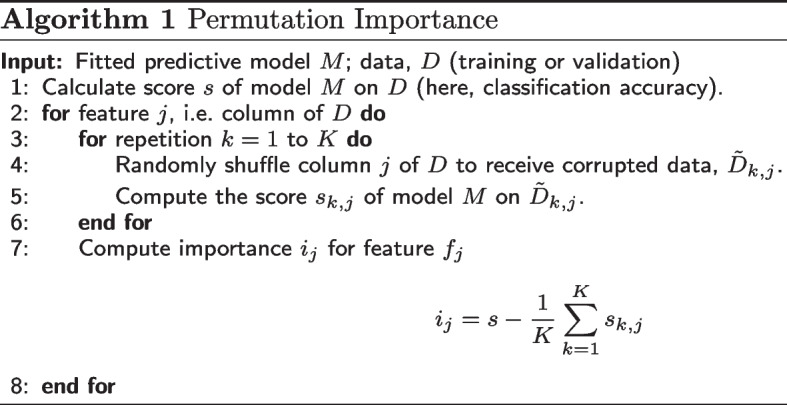


### Node features

The node-level features selected are defined as follows for node *i* (letting *w* be the number of nodes in the same community as *i*). Let the graph be $G = (V, E)$ with *V* denoting the set of nodes and *E* the set of edges:

- *Degree*: The number of edges adjacent to *i*, deg(*i*).
- $E_{in}$: The number of edges adjacent to *i* within its community. Adapted to a single node from the original definition by Radicchi et al. (2004)
- $E_{out}$: The number of edges adjacent to *i* which connect it to nodes outside its community. Adapted to a single node from the definition by Radicchi et al. (2004). Note that $E_{in} + E_{out} =$ deg(*i*).
- $E_{in}$ *over* $E_{out}$: For a given node, the ratio of the number of edges connecting it to other nodes within the same community, relative to the number of edges it has to nodes in other communities:
  $$\begin{aligned} \frac{E_{in}}{E_{out}}. \end{aligned}$$
- *Out Degree Fraction (ODF)*: Adapted to a single node from the definition by Flake et al. (2000), this is the ratio of edges connecting node *i* to nodes in other communities, relative to its total degree:
  $$\begin{aligned} \frac{E_{out}}{\mathrm{deg}(i)} \end{aligned}$$
- *Expansion*: Adapted from the definition by Radicchi et al. (2004), this is number of edges from a single node *i* to nodes assigned to other communities, normalised with respect to the number of nodes in the same community as *i*:
  $$\begin{aligned} \frac{E_{out}}{w} \end{aligned}$$
- *Cut Ratio*: Adapted to a single node from the graph cut measure discussed by Fortunato (2010). As with the metric above, this considers the number of outgoing edges from *i* to other communities, but in this case normalised with respect to the number of nodes **not** in the same community as *i*:
  $$\begin{aligned} \frac{E_{out}}{|V|-w} \end{aligned}$$
- *Conductance*: Adapted to a single node from the clustering objective described by Shi and Malik (2000), this measure is the ratio between the connections for node *i* within its community and its total number of connections:
  $$\begin{aligned} \frac{E_{out}}{\mathrm{deg}(i) + E_{in}} \end{aligned}$$
- *Average Shortest Path*: The mean of the shortest path lengths from node *i* to all other nodes in the graph.
- *Triangle Participation*: Let $c_i$ be the number of nodes with which node *i* shares a common neighbour within its assigned community. Then triangle participation is given by the fraction:
  $$\begin{aligned} \frac{c_i}{w} \end{aligned}$$
- *Clustering Coefficient*: The local clustering coefficient of a node measures how close its neighbours are to forming a clique (Watts and Strogatz 1998). Formally, let $T_i$ be the number of triangles containing *i* across the whole graph. Then the clustering coefficient of node *i* is given by:
  $$\begin{aligned} \frac{2T_{i}}{\mathrm{deg}(i)(\mathrm{deg}(i)-1)} \end{aligned}$$
- *Betweenness Centrality*: Let $\sigma (j,k)$ be the number of shortest (*j*, *k*) paths, and $\sigma (j,k|i)$ be the number of those paths that pass through *i*. Then the betweenness centrality of node *i* is given by Brandes (2001):
  $$\begin{aligned} \sum _{j,k \in V } \frac{\sigma (j,k|i)}{\sigma (j,k)} \end{aligned}$$
  Note that if $j = k$, $\sigma (j,k) = 1$ and if either *j* or $k = i$, then $\sigma (j,k|i) = 0$. Intuitively, a high betweenness centrality score for a node often indicates that it holds a bridging position in a network.
- *Eigenvector Centrality*: Proposed by Bonacich (1986). The eigenvector centrality of node *i* is the *i*th entry in the vector ${\textbf {x}}$ which solves the eigenvector equation:
  $$\begin{aligned} {\textbf {Ax}} = \lambda {\textbf {x}} \end{aligned}$$
  where ${\textbf {A}}$ is the adjacency matrix with node *i* represented in the *i*th row/column. Based on the definition above, this measures deems that a node is important if it is connected to other important nodes.
- *Closeness Centrality*: Refers to the centrality measure proposed by Freeman (1979). Let *d*(*i*, *j*) be the length of the shortest path between nodes *i* and *j*. Then the closeness centrality of node *i* is given by:
  $$\begin{aligned} \frac{|V|-1}{ \sum _{j \ne i} d(j,i)} \end{aligned}$$
  This provides us with an assessment of the extent to which node *i* is close to all other nodes in a network, either directly or indirectly.

### Node-pair features

Given a pair of nodes (*i*, *j*), we define a number of node-pair-level features:

- *Shortest Path Length*: The least number of edges separating nodes *i* and *j*.
- *Common Neighbours*: The number of shared nodes adjacent to both *i* and *j*, which we denote as $n_{ij}$.
- *Max Edge Centrality*: The maximum over centralities of all edges along the shortest path. The edge centrality is defined in a similar manner to betweenness centrality for nodes (Brandes 2001). That is, for a given edge *e*, we compute
  $$\begin{aligned} \sum _{j,k \in V } \frac{\sigma (j,k|e)}{\sigma (j,k)} \end{aligned}$$
  where $\sigma (j,k|e)$ now refers to the number of shortest paths between *j* and *k* passing through an edge *e* rather than a node *i*.
- *Cosine Similarity*: Frequently used to measure similarity for textual data, but can also be applied to assess node-pair similarity in the context of graphs:
  $$\begin{aligned} \frac{n_{ij}}{\sqrt{\mathrm{deg}(i)}\sqrt{\mathrm{deg}(j)}} \end{aligned}$$
- *Jaccard Coefficient*: A common set similarity measure, originally proposed in Jaccard (1912). In a graph context, let $\Gamma (i)$ be the set of neighbours of node *i*. Then the Jaccard coefficient of nodes *i* and *j* is given by:
  $$\begin{aligned} \frac{|\Gamma (i) \cap \Gamma (j)|}{|\Gamma (i) \cup \Gamma (j)|} \end{aligned}$$
  A higher value for this measure indicates a greater level of overlap between the neighbours of *i* and *j*, relative to their full sets of individual connections.

### Classification problems

For node-pair-level features, there is an obvious binary classification problem where pairs of nodes are labelled as belonging to the “same community” or to “different communities”. Since the algorithms of interest in our work are stochastic in nature, a pair of nodes may sometimes be in the same community, while for other runs of the algorithm the pair may not appear in the same community. Over the course of many runs of a given algorithm, pairs can simply be labelled as “same community” if they are in the same community for more than half of the runs, and “different community” if they are in the same community for less than half of the runs. In the unlikely event they are in the same community for exactly half of the runs, we have chosen arbitrarily to label them “same community”.

For node-level features, defining a classification problem is harder since, on consecutive runs of the community detection algorithm, the number of communities can vary, or the community labels can be permuted. Thus, classifying a node into its “correct” community is not a well-defined problem. Instead, we propose a binary classification problem determining whether the node is “easy” or “hard” to assign to a community, by observing how frequently it flips between communities on successive algorithmic runs. To define this mathematically, we require a coassociation matrix, described in “Coassociation matrix” section below. This will allow us to identify features that are predictive in whether a node is strongly associated with a specific community (near its “centre”), or whether it lies on the border between two or more communities. Nodes of the latter type may be of particular interest in certain domains, such as public health.

In order to label the nodes as “easy” or “hard” to assign to a community, we incorporate the use of a coassociation matrix, defined below.

### Coassociation matrix

For a given graph and community detection algorithm, we can construct a *coassociation matrix*, *C*, using the outputs of many runs of the algorithm on the graph. In our methodology, we use the same set of runs to calculate both the community-dependent features, and the coassociation matrix. Let $r_{ij}$ be the number of runs for which nodes *i* and *j* are in the same community, and let *R* be the total number of runs. The value for the entry *ij* in the matrix is given by:

$$\begin{aligned} C_{ij} = \frac{r_{ij}}{R} \end{aligned}$$

Intuitively, the coassociation matrix represents the proportion of runs for which two nodes are in the same community, for every pair of nodes.

In order to classify nodes as either “easy to cluster” or “hard to cluster”, we then calculate the entropy of each node from the coassociation matrix as follows:

$$\begin{aligned} E_{i} = \frac{\sum _{j} p_{ij}}{N} \end{aligned}$$

where *N* is the number of nodes and $p_{ij}$ is defined as follows:

$$\begin{aligned} p_{ij} =\left\{ \begin{array}{ll} -C_{ij}\log _{2}(C_{ij}) & \text{ if } C_{ij} > 0 \\ 0 & \text{ if } C_{ij} \le 0 \end{array}\right. \end{aligned}$$

Unfortunately, these entropy values are not as intuitively understood as the raw coassociation matrix entries. Thus, it is not as simple to label nodes as “easy to cluster” or “hard to cluster” directly from their entropy values as it is to label pairs as “same community” or “different community” directly from the coassociation matrix. Instead, once every node is assigned an entropy, we use one-dimensional *k*-means clustering (with $k=2$ clusters) to separate nodes into two training classes: those with low entropy belong to the “easy to cluster” class, and those with high entropy belong to the “hard to cluster” class. Intuitively, these correspond to nodes which are often assigned to the same community by the algorithm and those which are often assigned to different communities.

### Summary

Our aim is to identify human-interpretable graph features which relate to the community membership determined by a community finding algorithm. In order to select the more informative features from a predefined longlist of candidates, we define two simple binary classification problems: one for node-level features, where we will predict a node’s ease of assignment to a community; and one for node-pair-level features, where we will predict whether the two nodes belong to the same community or not. We will then find the permutation importance of each feature from our model to identify which features provide the most information about the output label.

## Methodology

Our experiments take place on more than one graph, $\mu$ value (as described in “Community finding” section), algorithm, and even classification task. Having several independent variables enables us to answer the following research questions:

*RQ1: Do the most informative node features depend on the community finding algorithm used?*

*RQ2: Do the most informative node-pair features depend on the community finding algorithm used?*

*RQ3: How do the most informative node features vary with the degree of community separation, as defined by the mixing parameter,* $\mu$*?*

*RQ4: How do the most informative node-pair features vary with the degree of community separation, as defined by the mixing parameter*, $\mu$*?*

*RQ5: In all cases, what are the most predictive features?*

Although we did not form a strong hypothesis for the latter three questions, we hypothesise that the most predictive features would vary by algorithm.

*H1: The most informative node and features will depend on the community finding algorithm.*

*H2: The most informative node-pair and features will depend on the community finding algorithm.*

**Fig. 1:**
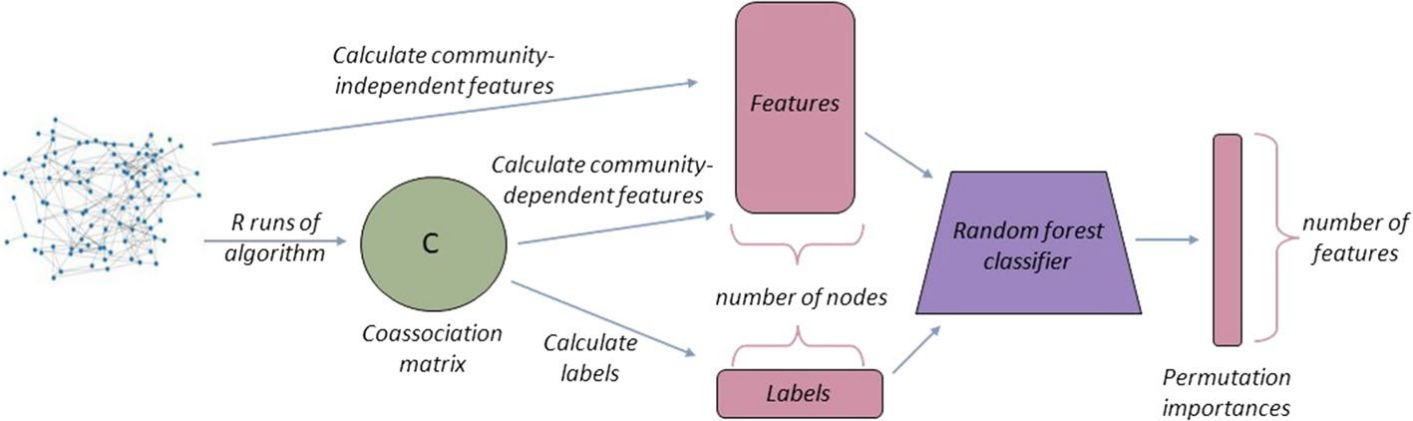
Experiments for determining explainable social network analysis metrics in the node feature experiment. A similar methodology is applied to the node-pair experiment. After *R* runs of the algorithm, a coassociation matrix is constructed encoding how often two nodes are classified in the same community. Feature values are computed and provided as input to a random forest classifier to determine permutation importance. The distributions of permutation importance can be compared across all graphs to identify explainable metrics

In order to answer the questions above, we now present our experimental and statistical methodology, which may prove useful in tackling the evaluation of community finding algorithms more generally. This methodology is also illustrated in Fig. 1.

### Experimental methodology

We test our approach using three popular methods to detect community structure, each based on different concepts (for example, we only use one modularity optimization algorithm):

1. *Infomap* (Rosvall and Bergstrom 2008), also known as the map equation, uses an information-theoretic approach, where nodes are represented by codewords composed of two parts, the first of which is provided by the community it belongs to. The community memberships are optimised by minimising the average code length describing random walks on the network.
2. *Louvain* (Blondel 2008), is a modularity optimization approach which involves two steps. Firstly, the common modularity objective is optimized at a local level to create small communities. Next, each small community is treated as a single node and the first step is repeated. By following this agglomerative process, a hierarchy of communities is constructed.
3. *LPA*, the label propagation algorithm proposed by Raghavan et al. (2007), assumes that nodes should belong to the same community as most of their neighbours. To begin, each node is initialised with a unique community and then these labels are then iteratively propagated through the network. After each iteration, a node receives the same label as the majority of its neighbours. Once this process is complete, nodes sharing the same label are grouped together as communities.

**Fig. 2:**
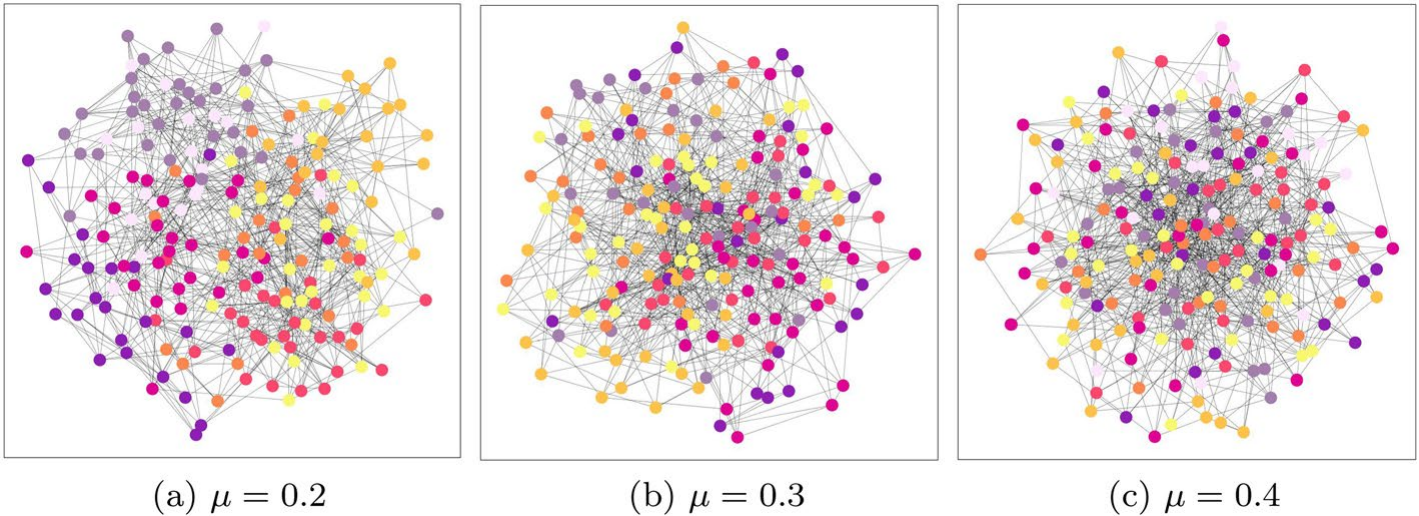
Example graphs with 200 nodes at the three $\mu$ values. Communities shown with colour. Increased mixing parameter increases the prevalence of edges between communities

When constructing our networks, we selected $\mu$ values of 0.2, 0.3, and 0.4. As described in “Community finding” section, this parameter controls the level of separation or mixing between communities, where the higher the value of $\mu$, the less easy it is to distinguish between different communities (Fig. 2).

At each value of $\mu$, a set of graphs, $\Gamma$, are generated before any experiments take place. This set of graphs is the same size, $|\Gamma |$, for each value of $\mu$. In order to match the hyperparameters used by Lancichinetti et al. (2008) in the original LFR benchmark paper, we use the LFR generator in NetworkX to generate networks with 1000 nodes of average degree 20 and maximum degree 50. We set the hyperparameters $\tau _1$ and $\tau _2$ to 3 and 2 respectively.

Each experiment is then defined by three categories: the $\mu$ value; the community detection algorithm; and the feature type (node vs node-pair). This results in 18 possible experiments from the 3 algorithms, 3 mixing parameters and 2 feature types. Data from the $|\Gamma |$ graphs at the relevant value of $\mu$ are used for the experiment. For each $\mu$-algorithm-feature type combination, the following procedure is then performed.

Firstly, the algorithm is run 1000 times on each of the $|\Gamma |$ graphs. Using these runs, any community-dependent features are calculated, along with the coassociation matrix. Features which are community-independent are also calculated at this stage, although they do not depend on the runs. The nodes or pairs-of-nodes must then be labelled according to the binary classification problem. The labelling procedures are described separately for the two feature-types in the relevant experiment sections.

Now, for each of the graphs of the experiment, we have a dataset of either nodes or pairs of nodes, each member of which is labelled and has a list of feature values. A random forest with 100 trees is then trained to classify the dataset for the specific graph.

During training we use 5-fold cross-validation and repeat this for 10 training runs. A permutation importance is calculated for each node or node-pair feature after each of the 50 runs, using the held-out test data. At the end of the 50 cross-validation runs, a mean average of the 50 gathered permutation importance scores is taken for each node or node-pair feature. This gives us its final importance score as generated by this graph. Overall, this results in $|\Gamma |$ permutation importance values for each feature. The full experimental methodology for node features is represented in Algorithm 2. For node-pair features, the algorithm is identical, looping over node-pairs instead of nodes.
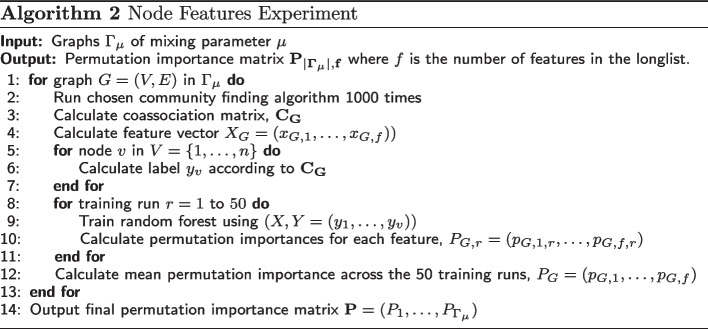


### Statistical methodology

For both experiments above, we have distributions of our features over the runs of the experiment. These distributions can be compared to determine statistical significance of the difference between them, and the size of this difference, in order to identify the features of interest. This statistical analysis and the final conclusions drawn are specific to the $\mu$-algorithm-feature type combination of the experiment.

In order to develop an appropriate statistical methodology, we performed a pilot study using 20 graphs at each $\mu$ value (giving 60 graphs in total). For each experiment, this gave us 20 values of permutation importance for each feature, on which we carried out Shapiro-Wilk tests. In this pilot study, $67\%$ of the features across all algorithms, feature types and $\mu$ values were normally distributed, so we started with a normal assumption. On this basis, the statistical methodology would be as follows:

1. Perform a power analysis with a normal assumption to determine the value of $|\Gamma |$ required to draw statistically significant conclusions.
2. Carry out the experiments to obtain $|\Gamma |$ values for each feature of each experiment.
3. Confirm with a repeat of the Shapiro-Wilk tests that these $|\Gamma |$ values are indeed normally distributed in the majority of cases.
4. If the distributions are normal, perform pairwise t-tests with Bonferroni–Holm corrections using these values. Otherwise, perform pairwise Wilcoxon tests with Bonferroni–Holm corrections using these values.

Power analysis was conducted with the following parameters: Cohen’s effect size of 0.3, significance level of 0.05, and a power of 0.9. The power analysis concluded 119 graphs were necessary for our experiment, which we rounded to 120. At this stage, we generated 360 new graphs (120 at each $\mu$ level) for our experiment.

The application of this methodology to our new data set revealed that the distributions of metric values were not normally distributed. Therefore, to determine significance, pairwise Wilcoxon tests with Bonferroni–Holm correction were applied to our data to determine the significant results.

## Exp. 1: node feature experiment

**Table 1:** The numbers of communities identified by each algorithm on graphs with 1000 nodes

Number of communities			
	Mean	Median	Std.
Infomap, mu 0.2	40.65	41.00	2.45
Infomap, mu 0.3	39.93	40.00	2.33
Infomap, mu 0.4	35.76	36.00	2.65
Louvain, mu 0.2	34.45	34.00	1.83
Louvain, mu 0.3	29.56	29.00	1.79
Louvain, mu 0.4	24.20	24.00	1.61
LPA, mu 0.2	39.14	39.00	2.54
LPA, mu 0.3	36.33	36.00	3.06

The mean, median and standard deviation are calculated across all 120 graphs in each case

**Table 2:** Statistics on the normalised mutual information scores for two algorithms on graphs of the same $\mu$ value

NMI statistics			
	Mean	Median	Std.
Infomap and Louvain, mu 0.2	0.977	0.978	0.008
Infomap and Louvain, mu 0.3	0.949	0.949	0.012
Infomap and Louvain, mu 0.4	0.883	0.884	0.021
Infomap and LPA, mu 0.2	0.991	0.992	0.007
Infomap and LPA, mu 0.3	0.962	0.966	0.022
Louvain and LPA, mu 0.2	0.970	0.970	0.012
Louvain and LPA, mu 0.3	0.922	0.925	0.025

For each row of the table, 1000 pairs of partitions were uniformly randomly chosen for each graph. As there are 120 graphs for each $\mu$ value, this means 120,000 values contribute to each statistic

Once the experimental data was collected, the 0.4-LPA-node and 0.4-LPA-node-pair experiments were omitted. This is because LPA clustered a majority of nodes into one large community at this $\mu$ value, generating features and labels that were not suitable for our experiments. Essentially, LPA was unable to recognise community structure at this high degree of mixing. All other experimental data is reported.

Tables 1 and 2 respectively show statistics on the number of communities detected by each algorithm across graphs of a common $\mu$ value, and normalised mutual information (NMI) scores comparing the performance of pairs of algorithms. In the first of these, we can see that communities range in number from 24 to 40, resulting in a mean community size between 25 and 45 nodes. In reality, sizes of communities created using the LFR generator follow a power law, so many will be much larger or smaller than the mean. In the second table, NMI scores are generally high in all cases, although decrease as the $\mu$ value increases, as one might expect. Overall these results suggest that there are some communities with very large, stable cores, and that the nodes which frequently change across multiple algorithmic runs are single nodes on the periphery of these large communities, or belong to the much smaller communities.

### Experiment

The classification labels for the node feature experiments are calculated for a single graph as follows. The entropy of each node is calculated from the coassociation matrix of the current graph, and *k*-means clustering of these entropy values is performed to separate the nodes into “easy to cluster” and “hard to cluster” nodes. However, using this process, we have a very low proportion of “hard to cluster” nodes. The proportion of nodes labelled as “hard to cluster” are reported in Table 3. For low mixing parameter values, this can be as low as $9\%$. This reinforces the finding from Tables 1 and 2 that there are large, central cores to the communities with a small number of nodes on the periphery or in smaller communities. However, the proportion of “hard to cluster” nodes can rise to as high as $25\%$ with an increased mixing parameter, indicating that this is a distinct class of nodes. Due to the low proportions of “hard to cluster” nodes, we propose using undersampling. Rather than undersampling randomly, we propose using the “easiest” nodes to cluster (those with the lowest entropy) until the number of “hard” nodes is $75\%$ that of the number of “easy” nodes. Using this *strategic undersampling* method enables us to identify node features which distinguish between truly separate classes, rather than distinguishing between nodes with an entropy either side of the arbitrary cut-off generated by the *k*-means clustering.

**Table 3:** Statistics on the proportion of nodes labelled as “hard to cluster” after running each algorithm on graphs of varying $\mu$ value

Proportion of “hard to cluster” nodes			
	Mean	Median	Std.
Infomap, mu 0.2	0.087	0.060	0.072
Infomap, mu 0.3	0.076	0.067	0.044
Infomap, mu 0.4	0.192	0.180	0.070
Louvain, mu 0.2	0.169	0.152	0.101
Louvain, mu 0.3	0.199	0.175	0.120
Louvain, mu 0.4	0.227	0.221	0.081
LPA, mu 0.2	0.242	0.250	0.117
LPA, mu 0.3	0.253	0.250	0.115

The mean, median and standard deviation are calculated across all 120 graphs in each case

### Results

**Fig. 3:**
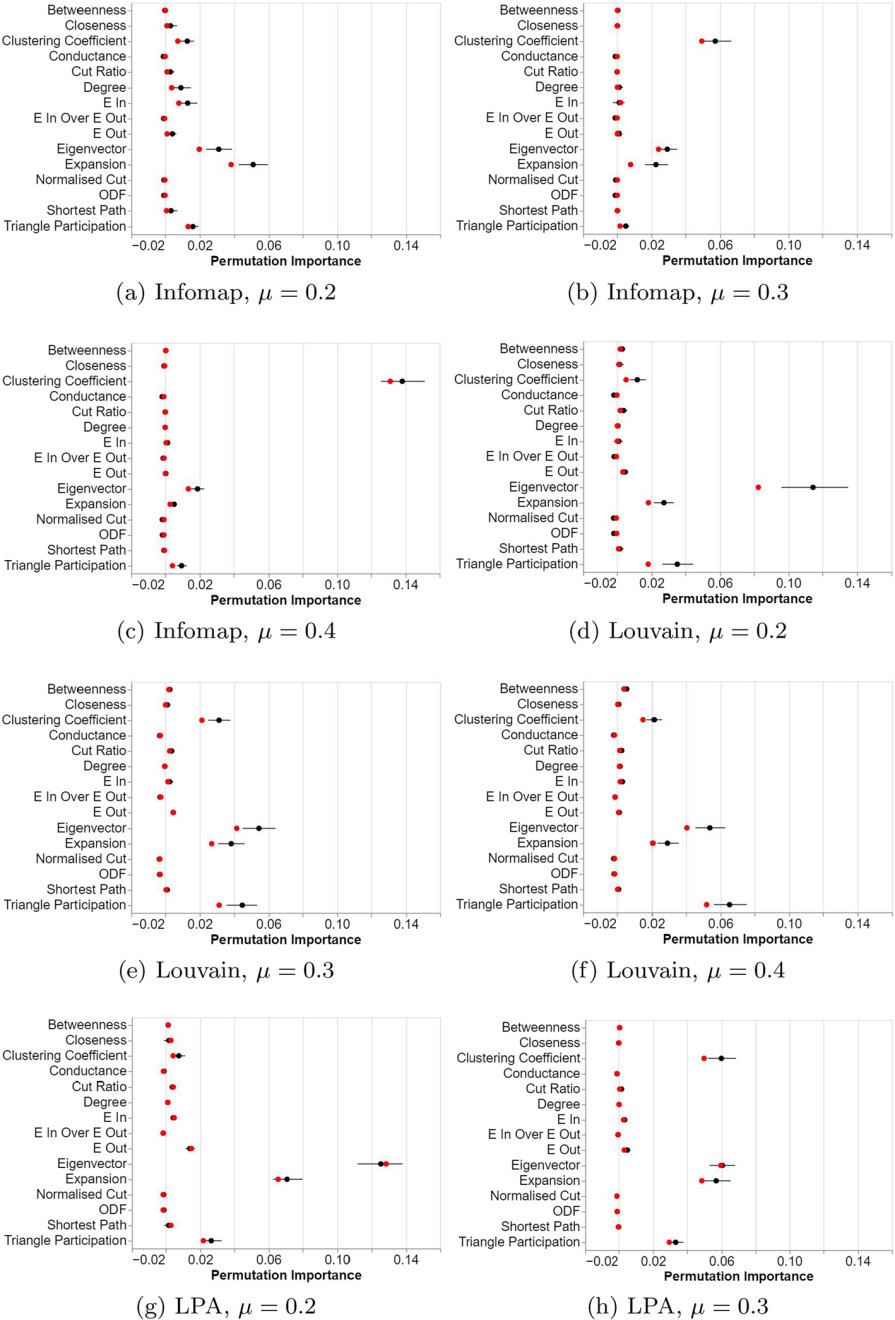
Results of the node feature experiments. Plots are of permutation importance of the metrics. Mean indicated as a black dot and median as a red dot. Lines indicate 95% bootstrapped confidence intervals

From these three experiments (displayed in Fig. 3), we see that four of the features consistently have a non-zero permutation importance: clustering coefficient, eigenvector centrality, expansion and triangle participation. We focus on reporting the significant differences for these features and provide full results in the supplementary material. Across all experiments at all $\mu$ levels, our pairwise Wilcoxon tests confirmed that these four features were significantly more important than the rest of the features, with the following exceptions:

- For Louvain at $\mu = 0.2$, clustering coefficient was not significantly different from betweenness centrality, cut ratio, or $E_{out}$.
- For Infomap at $\mu = 0.2$, clustering coefficient was not significantly different from degree, $E_{in}$, $E_{out}$ or shortest path. Triangle participation was not significantly more important than degree or $E_{in}$.
- For Infomap at $\mu = 0.3$, clustering coefficient was not significantly different from closeness centrality, degree, $E_{in}$ or shortest path.
- For LPA at $\mu = 0.2$, clustering coefficient was not significantly different from any of betweenness centrality, closeness centrality, cut ratio, degree, $E_{in}$ or average shortest path.

These exceptions align with what can be seen qualitatively: at the lowest $\mu$ level, some other features appear to be important such as degree, $E_{in}$ and perhaps even closeness centrality, cut ratio, $E_{out}$ and average shortest path. However, the effect size for all of these is much smaller than for the four most prominent features, and their significance vanishes at the two higher $\mu$ levels.

### Discussion

With respect to our research question RQ1 and in contradiction to our hypothesis H1, we observed that the four prominent features for predicting whether a node was difficult to classify did not depend on the algorithm used: clustering coefficient, triangle participation, eigenvector centrality, and expansion. In relation to the first two, this is not unexpected as these characteristics have previously been shown to be broadly indicative of good community structure (Harenberg et al. 2014). One could conjecture that nearly all community finding approaches would try to preserve cliques (accounting for the importance of clustering coefficient and triangle participation). In fact, cliques have often been used as seeds for constructing larger communities in the context of quite different community detection algorithms (Palla et al. 2005; Lee et al. 2010). Meanwhile, it seems reasonable that a node with many links to nodes in other communities relative to the number of nodes in its own community would be harder to classify, as it likely lies on the periphery of its community, close to one or more others (accounting for the importance of expansion).

At a surface level, the prominence of eigenvector centrality is more surprising, especially given the level of its performance. This centrality measure has similarities to PageRank (Page et al. 1999), where high values correspond to nodes at short distances from many high degree nodes. Nodes within a community’s core are more likely to have high degree and to be a short distance from other high degree nodes, with edges that connect other nodes within the community. The relationship between eigenvector centrality and regions of high density within the core versus periphery of a network was recently highlighted by Jayne and Bonacich (2021). Thus, in our case high values of eigenvector centrality might correspond to an increased chance that this node forms a part of the stable community core, rather than being an unstable node on a community’s periphery.

The results of our experiment with regards to changing mixing parameters $\mu$ (RQ3) indicate that these four features remain prominent. There is some evidence as well that the other features diminish in prominence as $\mu$ increases and the communities become more difficult to find. Thus, the same features are involved for explaining why a node is part of a stable core or changes communities between runs and all become statistically significant at higher mixing parameters.

Further investigation is required to find out why the important features consistently performed the best and the relative differences between them across other community finding algorithms.

## Exp. 2: pairwise community membership

As mentioned in “Exp. 1: node feature experiment” section, the 0.4-LPA-node-pair experiment is omitted here as LPA classified the entire graph as one community on a number of occasions at the higher mixing parameter level.

### Experiment

As with the node feature experiments, labelling all pairs directly as “same community” or “different community” results in imbalanced classes. However, we have vastly more data for the pairs of nodes than for the single nodes. Therefore, we propose undersampling both classes by randomly selecting the same number of “same community” and “different community” pairs from the available data. We choose to undersample randomly here rather than “strategically” since there are no pairs of nodes close to the threshold of 0.5 between “same” and “different” community, but choosing the highest and lowest values leads to a classification problem which is *too* easy. We select 1000 training examples for each class.

### Results

**Fig. 4:**
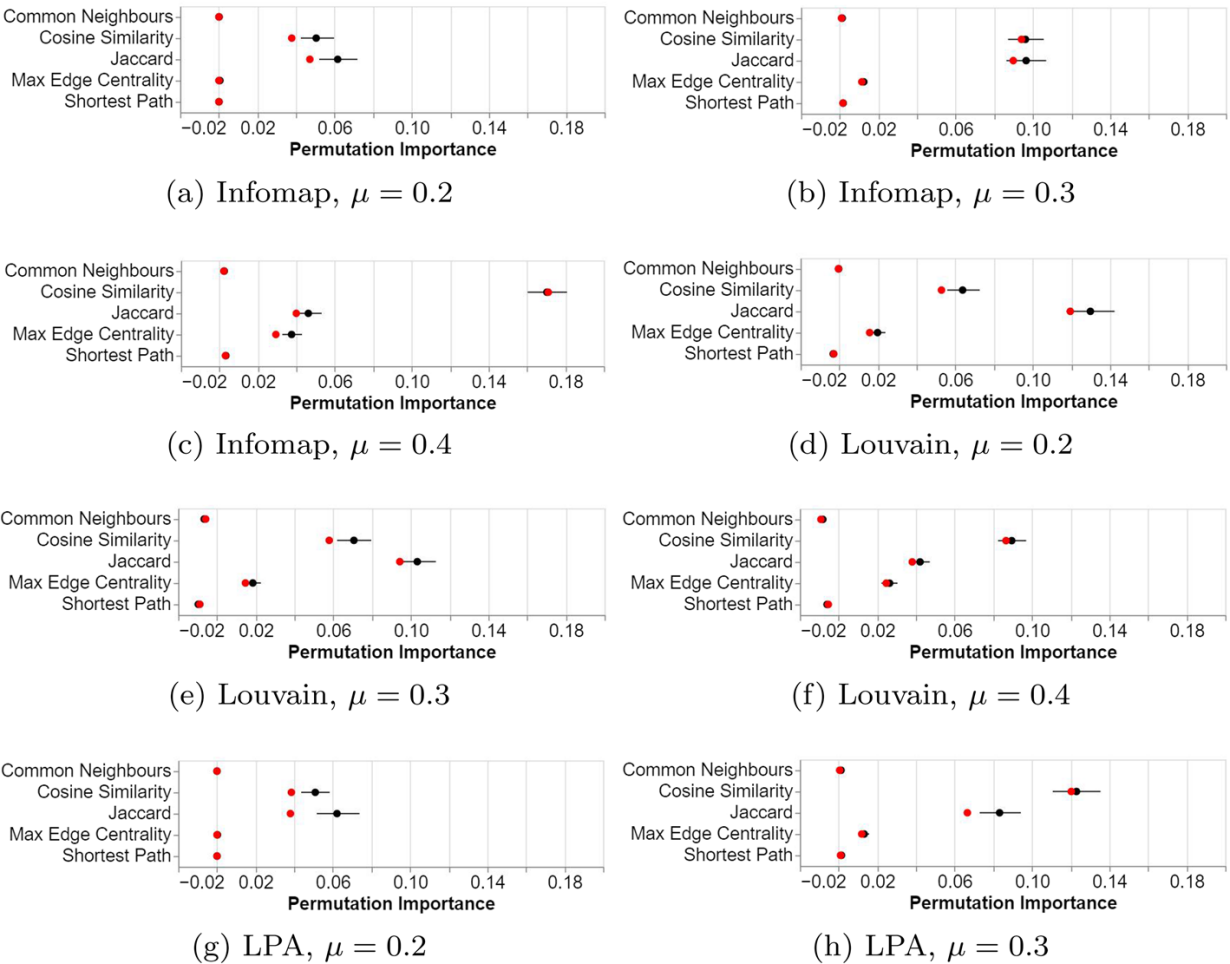
Results of the pair feature experiments. Plots are of permutation importance of the metrics. Mean indicated as a black dot and median as a red dot. Lines indicate 95% bootstrapped confidence intervals

As with our previous experiment, we found that two features were consistently important across the three community finding algorithms: cosine similarity and the Jaccard coefficient (displayed below in Fig. 4). We also found that the maximum edge centrality along the shortest path became more important at higher mixing parameter levels. This varied a little by algorithm; for Louvain it became important even at the lowest mixing parameter level of 0.2, however for Infomap and LPA it didn’t become important until the mixing parameter of 0.3.

We focus on the significant difference between these features. For a complete set of results, we refer to the supplementary material. The pairwise Wilcoxon tests confirmed that all three were significantly different across all experiments, including for max edge centrality at the $\mu = 0.2$ level despite the small effect size.

### Discussion

In contradiction to hypothesis H2, all algorithms performed similarly, with the most important features being Jaccard and cosine similarity. Both features compare the neighbourhoods of the selected nodes. Their importance is supported by the *local consistency assumption* (Zhou et al. 2004), frequently discussed in the context of instance-based classification, which asserts that neighboring instances will frequently share similar characteristics. In the context of unsupervised community detection, this corresponds to neighbouring nodes belonging to the same community, rather than having the same class label. This result is also congruous with the result of the first experiment, which found similar features to be important.

In response to RQ4, maximum edge centrality proved increasingly important as the $\mu$-level of the generated data increased. This measure, which is the maximum edge centrality measure along the shortest path between the two nodes, could be indicative of important edges that bridge two communities, i.e. the weak ties, and has been used in the past for divisive methods of community detection (Girvan and Newman 2002). The increased importance at higher values for the mixing parameter could be explained by how important local information is to determining if two nodes are within the same community. If $\mu$ is low, communities are well separated and local information almost completely describes if two nodes are in the same community. However, as $\mu$ increases, the value of local information decreases in importance. Instead, global information, such as determining if the path between the two nodes likely contains an edge that lies between communities, becomes critical in determining whether nodes belong to the same community.

Further investigation is required to find out why these features consistently performed the best and the relative differences between them across other community finding algorithms.

## Discussion and limitations

### General results discussion

Our hypothesis for both experiments was that the important metrics for would be dependent on the community finding algorithm, however, the same metrics were identified consistently. This indicates that there are common metrics that can be used to explain these phenomena, at least when producing explanations on the same dataset for the three algorithms tested. As our study is limited to networks generated by the LFR algorithm, the common metrics of importance could be indicative of structure produced by this method. Further work should be carried out on other datasets to see how this affects the metrics of importance. If the community finding algorithm can be taken into account, then these important metrics can also be weighted in a way that is in line with the algorithm and degree of mixing of the communities.

To validate the results of these experiments, we also ran them using Shapley values in place of permutation importance. Shapley values are well known among the explainability community, and are known to have mathematically desirable properties for producing explanations. Since our power analysis was performed using values of permutation importance, we report these as our main results, and report the results with Shapley values in the supplementary material.

In the case of the node experiments, we see clearly that for Louvain and LPA, the same four node features are more important with increasing mu value for Shapley values as we saw with permutation importance: clustering coefficient, expansion, eigenvector centrality, and triangle participation. With Infomap, there is some variation; the same four features are seen to be important, though not at all mu values. In addition, E In is shown to be important, as are Degree and Closeness Centrality at the lower mu values.

In the case of the node-pair experiments, the same trends are seen for Shapley values as for permutation importance across all algorithms. Jaccard and cosine similarity are consistently the most important, with max edge centrality increasing in importance with rising mu value.

The features used in both experiments, which are well understood by the network analysis community, can be used to gain a greater understanding of community structure in online social networks. For public health applications, these metrics may be used to understand phenomena such as social contagion (Brown et al. 2017; Valente and Yon 2020) and to plan interventions (Valente 2012), but their practical use remains future work. In future studies, we plan on integrating visualisation methods and performing studies to ensure that approaches such as the ones proposed here have impact in explaining network phenomena to address real-world user needs. We envisage these results could be used with a visualisation system where the communities assigned by an algorithm can be explored by selecting individual nodes or pairs of nodes to understand their community assignment. When a node flips between different communities on consecutive runs of the community finding algorithm, important feature values such as those identified in this work could be visually reported and compared relative to other nodes in the same or in different communities for further study by an expert.

Consensus clustering (Lancichinetti and Fortunato 2012b) is a way of dealing with nodes that are difficult to classify: run the community finding algorithm many times and determine the average result of these runs. Given that this study indicates that the metrics to determine if nodes are easy or hard to cluster by community finding algorithms are consistent across algorithm, the results of consensus clustering approaches could be augmented with these metrics to help determine which community these nodes should be clustered into, but this remains an area of future work. Also, values for these metrics could be used to seed the stable core of a community and then find other nodes that are less easy to cluster. This approach could lead to other partitioning algorithms or potentially overlapping community finding algorithms where “hard to cluster” nodes are partially contained by multiple communities. However, the effectiveness of such an approach would still need to be evaluated.

Although partitioning algorithms are usually used to find communities in networks in public health settings, overlapping community finding algorithms (Lancichinetti et al. 2011; Pallaand et al. 2005) can assign a single node to multiple communities. The studies that we present here suggest metrics used in social network analysis can be used to explain partitioning algorithms. In future work, it would be interesting to see if these, or other metrics, extend to overlapping community finding.

### Limitations and future work

Although there were benefits to our use of synthetic LFR data, these networks are ultimately an approximation for real data. As discussed previously, the use of real data for this analysis would have been tricky due to the lack of large datasets of networks with consistent structure over which we could draw statistically significant conclusions. Additionally, we would not have been able to vary parameters such as the mixing parameter $\mu$ to observe their effect on the results. Nevertheless, it is a limitation of this study that we are only able to confirm these results across a dataset of synthetic networks based on the parameters specified in an important community finding experiment (Lancichinetti and Fortunato 2009) on three community finding algorithms. In future work, the performance of these informative features could be verified on a smaller number of real networks, to investigate whether this affects the metrics of greatest importance. Even in the case where the metrics of greatest importance are heavily dependent on the dataset, the methodology presented here could beneficially be applied to new settings in order to gain insight into complex networks relevant to different applications, such as social, biological, and financial (Avdjiev et al. 2019; Giudici et al. 2017) networks.

Another area for future work would be to consider applying the more recent Shapley-Lorenz approach to explainability (Giudici and Raffinetti 2021) in place of using Shapley values or permutation importance; this approach is well-suited to settings such as ours, where the response variable is categorical.

## Conclusion

This paper presents the results of two experiments designed to move towards explainable community finding and explainable network analysis in general. Despite the different methods used by the algorithms in our study, consistent social network analysis metrics can be used to explain community structure in a post-hoc manner for these three algorithms on LFR networks. The results of our study indicate that commonly understood metrics used for network analysis can be used by an expert to explain community structure, bringing benefits to application areas where network data is prevalent, from computational social science (Lazer et al. 2009) to public health studies (Luke and Harris 2007).

## Supplementary Information

**Additional file 1.** Supplementary material is made available containing detailed descriptions and results plots of statistical analysis.

## Data Availability

Data and code implementations for the main experiments are available in the GitHub repository at https://github.com/sophiefsadler/community_finding. A zip file containing results and python scripts for statistical analysis can also be found on OSF at: https://osf.io/g4bwt/.
