# Supplementary material for: Towards explainable community finding
Source: Appl Netw Sci. 2022 Dec 8;7(1):81. doi: 10.1007/s41109-022-00515-6 (PMC9731939; doi:10.1007/s41109-022-00515-6)
Supplement: Supplementary file 1 — Additional file 1. Supplementary material is made available containing detailed descriptions and results plots of statistical analysis. [file 41109_2022_515_MOESM1_ESM.pdf]

RESEARCH

Towards Explainable Community Finding: Supplementary Material

Sophie Sadler[1*], Derek Greene[2*] and Daniel Archambault[1*]

*Correspondence:
1915996@swansea.ac.uk;
derek.greene@ucd.ie;
d.w.archambault@swansea.ac.uk
[1]Swansea University, UK
[2]School of Computer Science, University College Dublin, Ireland
Full list of author information is available at the end of the article

This supplementary material relates to the paper Towards Explainable Community Finding by Sadler et. al [1]. A zip file containing results and python scripts for statistical analysis can be found on OSF at: https://osf.io/g4bwt/

1 Feature Values

Figure 1, displayed below, shows the range of values the important features take for data points belonging to both classes in each of the classification problems. Subfigures (a) - (d) show feature values for nodes of both the "easy to cluster" and "hard to cluster" classes, while subfigures (e) - (g) show feature values for pairs of nodes belong to both the same and different communities. In all cases, the solid marks represent the minimum and maximum values of these features across all 120 graphs of the relevant mu level, while the translucent mark represents the mean value.

2 Pairwise Wilcoxon Tests

Figures 2 and 4 displayed below, also present in the paper, show distributions of the features' permutation importances. Each plot represents one algorithm run on 120 graphs of one μ value. Thus, for each feature on the y axis, there are 120 permutation importance values. The black and red circular marks represent the mean and median of these values, while the black bar represents a non-parametric bootstrap of the 95% confidence interval.

For each plot in figures 2 or 4, there is then a corresponding plot in figures 3 or 5 respectively. The plots in figures 3 and 5 are heatmaps representing the results from pairwise Wilcoxon tests between features. These tests were run for every pair of features to identify whether the distributions of the permutation importance values for the two features were significantly different (subject to Bonferroni-Holm corrections). The colour of the cell in the heatmap represents whether there was a significant difference or not, with a key in the upper right. A significance of 0 represents no significant difference; a significance of 1 represents a significant difference.

2.1 Node Feature Experiments

For the node feature experiments, we observed qualitatively from figure 1 that four of the features consistently have a non-zero permutation importance: clustering coefficient, eigenvector centrality, expansion and triangle participation. Our pairwise Wilcoxon tests confirmed, as reported in the main paper, that these four features

were significantly different from the rest of the features, with the following exceptions:

- For Louvain at $\mu = 0.2$, clustering coefficient was not significantly different from betweenness centrality, cut ratio, or E_{out}.
- For Infomap at $\mu = 0.2$, clustering coefficient was not significantly different from degree, E_{in}, E_{out} or shortest path. Triangle participation was not significantly more important than degree or E_{in}.
- For Infomap at $\mu = 0.3$, clustering coefficient was not significantly different from closeness centrality, degree, E_{in} or shortest path.
- For LPA at $\mu = 0.2$, clustering coefficient was not significantly different from any of betweenness centrality, closeness centrality, cut ratio, degree, E_{in} or average shortest path.

This can be observed from the heatmap in figure 2.

2.2 Pair-node Feature Experiments

For the pair-node feature experiments, we observed qualitatively from figure 4 that two features were consistently important across the three community finding algorithms: cosine similarity and the Jaccard coefficient. We also found that the maximum edge centrality along the shortest path became more important at higher mixing parameter levels. The pairwise Wilcoxon tests, as displayed in figure 5, confirmed that all three were significantly more important across all experiments, including for max edge centrality at the $\mu = 0.2$ level despite the small effect size.

3 Shapley Values

The node and node-pair experiments were also carried out with the use of Shapley values in place of permutation importance, in order to verify the results with an alternative importance ranking method. The same experimental data was used, with the only difference being the calculation of feature importance. These results are displayed in figures 6 and 7.

In the case of the node experiments, we see clearly that for Louvain and LPA, the same four node features are more important with increasing mu value as we saw with permutation importance: clustering coefficient, expansion, eigenvector centrality, and triangle participation. With Infomap, there is some variation; the same four features are seen to be important, though not at all mu values. In addition, E In is shown to be important, as are Degree and Closeness Centrality at the lower mu values.

In the case of the node-pair experiments, the same trends are seen as for permutation importance. Jaccard and cosine similarity are consistently the most important, with max edge centrality increasing in importance with rising mu value.

4 Results Files

The zip file contains a sub-folder for each of the three algorithms. Within each of these is a pickle results file for each of the graphs on which this algorithm was run. Each results file contains the graph number (1-120), the μ value (0.2, 0.3, 0.4 represented by 2, 3, 4) and the type of experiment (node, pair) in the title. For example, for graph 11 at a μ value of 0.3 on the node features, the file is named: "graph_11_mu_0_3_node" and can be loaded in Python like so:

```
import pickle
with open('graph_11_mu_0_3_node', 'rb') as f:
    results = pickle.load(f)
```

The results object is then a Python dictionary. The keys and values of the results dictionary are as follows:

- *Stable Nodes*: Present in node experiments only. Int representing number of stable nodes.
- *Unstable Nodes*: Present in node experiments only. Int representing number of unstable nodes.
- *Different Communities*: Present in pair-node experiments only. Int representing number of pairs of nodes in different communities.
- *Same Communities*: Present in pair-node experiments only. Int representing number of pairs of nodes in the same community.
- *Stability Cutoff*: Present in node experiments only. Float representing entropy value below which nodes are labelled stable, and above which nodes are labelled unstable. This cutoff is determined through one dimensional k-means $k = 2$.
- *Undersampling Level*: Float representing amount of undersampling.
- *Feature Importances*: Dictionary. Keys are the feature names as displayed on plots below. Values are floats representing the permutation importance of the feature for this random forest.
- *Accuracy Scores*: List of 50 floats representing accuracy on 50 cross-validation runs.
- *Balanced Accuracy Scores*: List of 50 floats representing balanced accuracy on 50 cross-validation runs.

5 Analysis Scripts

Also included in the zip file are three Python scripts containing the code used for our statistical analysis. As long as these are in the same location as the three algorithm sub-folders containing the results, no adjustments to the code are necessary and they can be run immediately to obtain a statistical output.

The first of these is the "shapiro_tests.py" file. Running this will generate results from Shapiro tests used to identify if the permutation importances are normally distributed, in two new CSV files: "node_feature_shapiro_vals.csv" and "pair_feature_shapiro_vals.csv". Each of these contains three columns: an index column, a column titled "Shapiro" and a column titled "p_Value". The values in the index column are named with the algorithm, μ value and feature, for example: "Louvain_mu_0_2_Degree". Due to the presence of the index column, the CSV file must be loaded in Python with the index_col flag:

```
pd.read_csv('node_feature_shapiro_vals.csv',
index_col=0)
```

The "Shapiro" and "p_Value" columns then contain the value of the Shapiro statistic and the p value for the feature named in the index column.

The second script is the "Wilcoxon_tests.py" script. This generates the heatmaps displayed below showing which pairs of features have significantly different permutation importance distributions. Heatmaps will be stored in a new folder

entitled "Results_Plots". The script also generates an accompanying CSV file, "wilcoxon_vals.csv" containing p values from the Wilcoxon tests. As with the CSV files produced by the Shapiro test script, this has 3 columns, the first of which is an index column naming the two features which are being compared. The second column is titled "Wilcoxon p Value" and contains the p value for that pair of features. The third and final column is titled "Compare to" and contains the value that this p value must be compared with to determine significance. This value varies, due to the Bonferroni-Holm correction.

The final script is the "distribution_plot_gen.py" file. This generates the distribution plots shown in figures 2 and 4 below. Implicitly calculated in this process are the mean, median and non-parametric bootstrap of the 95% confidence interval, as Altair carries out these calculations when generating the plots. As with the heatmaps, these will be saved in a "Results_Plots" folder.

Author details
[1]Swansea University, UK. [2]School of Computer Science, University College Dublin, Ireland.

References

1. Sadler, S., Greene, D., Archambault, D.: Towards explainable community finding. Applied Network Science (2022)

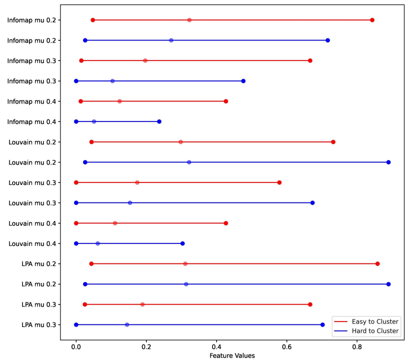

(a) Clustering Coefficient

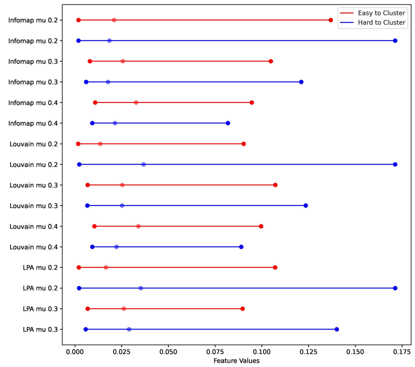

(b) Eigenvector Centrality

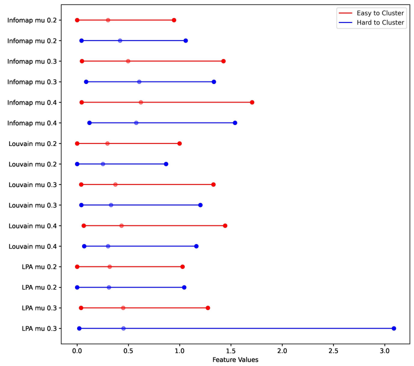

(c) Expansion

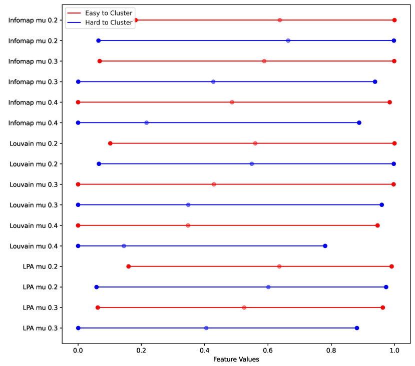

(d) Triangle Participation

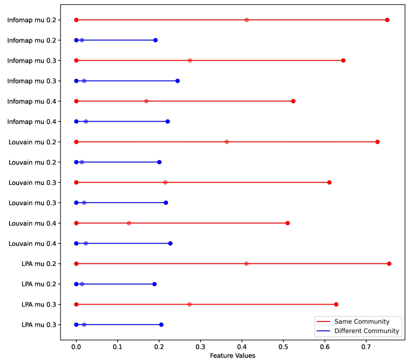

(e) Cosine Similarity

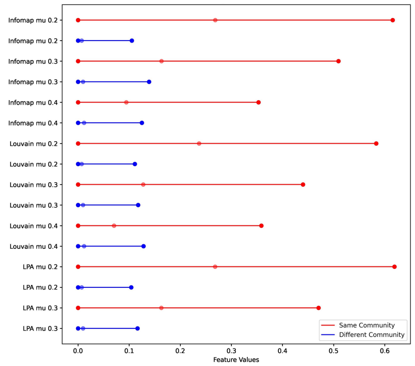

(f) Jaccard

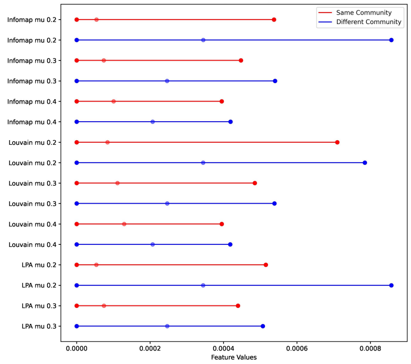

(g) Max Edge Centrality

Figure 1: Values of the most important features for both classes in each classification problem.

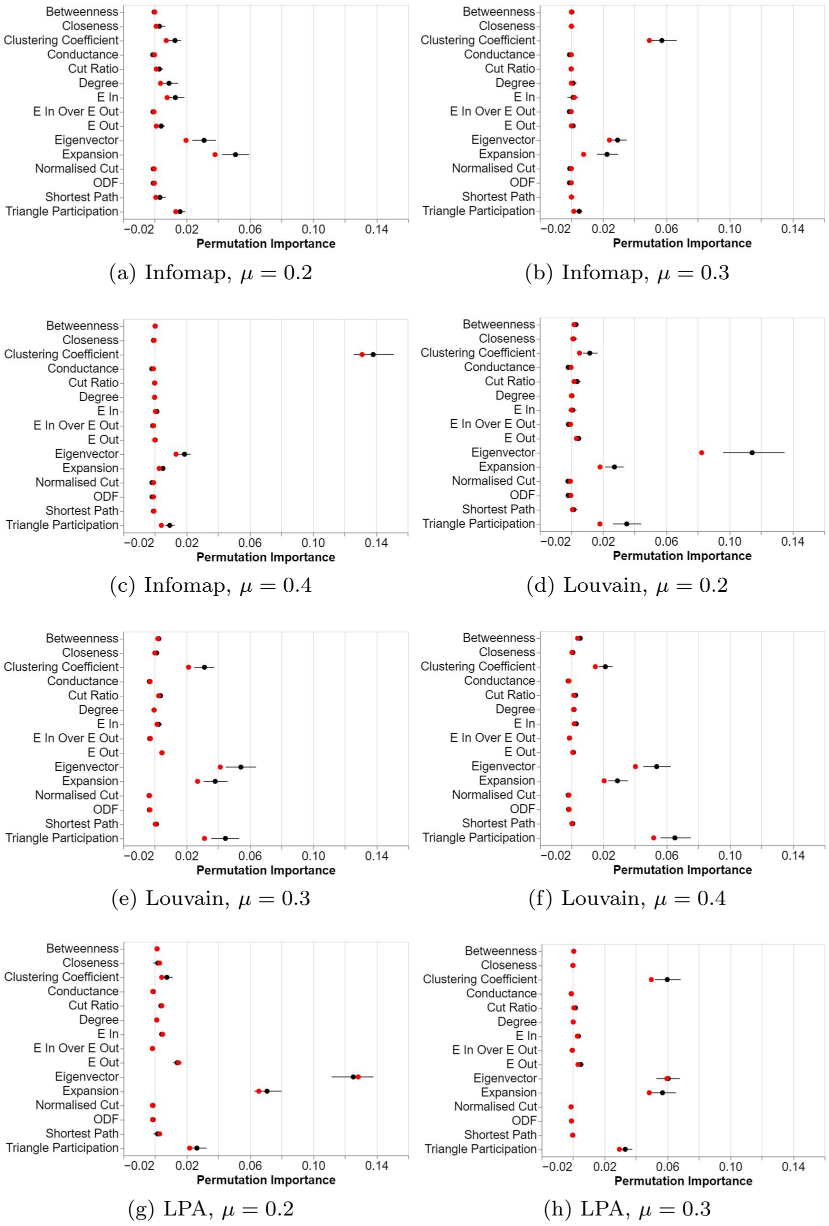

(a) Infomap, $\mu = 0.2$ (b) Infomap, $\mu = 0.3$

(c) Infomap, $\mu = 0.4$ (d) Louvain, $\mu = 0.2$

(e) Louvain, $\mu = 0.3$ (f) Louvain, $\mu = 0.4$

(g) LPA, $\mu = 0.2$ (h) LPA, $\mu = 0.3$

Figure 2: Results of the node feature experiments

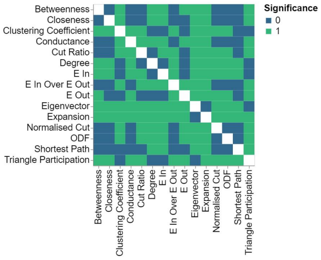

(a) Infomap, $\mu = 0.2$

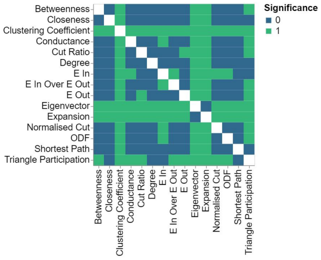

(b) Infomap, $\mu = 0.3$

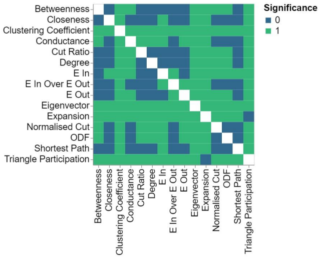

(c) Infomap, $\mu = 0.4$

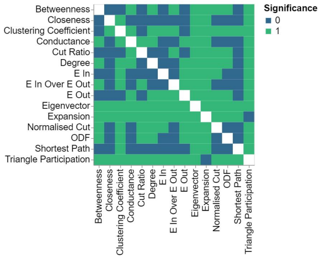

(d) Louvain, $\mu = 0.2$

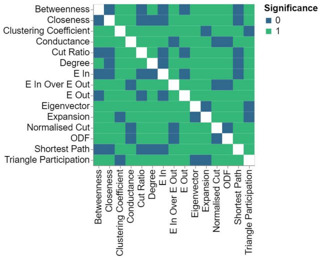

(e) Louvain, $\mu = 0.3$

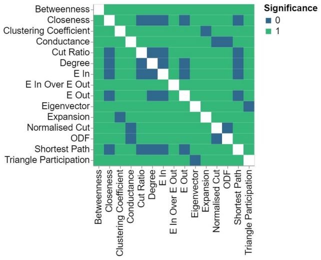

(f) Louvain, $\mu = 0.4$

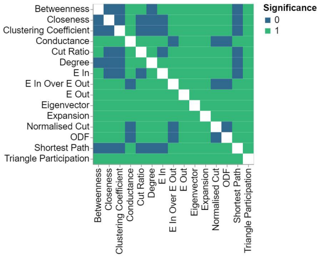

(g) LPA, $\mu = 0.2$

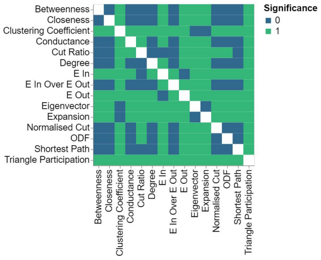

(h) LPA, $\mu = 0.3$

Figure 3: Heatmaps showing significance of Wilcoxon tests on the node feature experiments

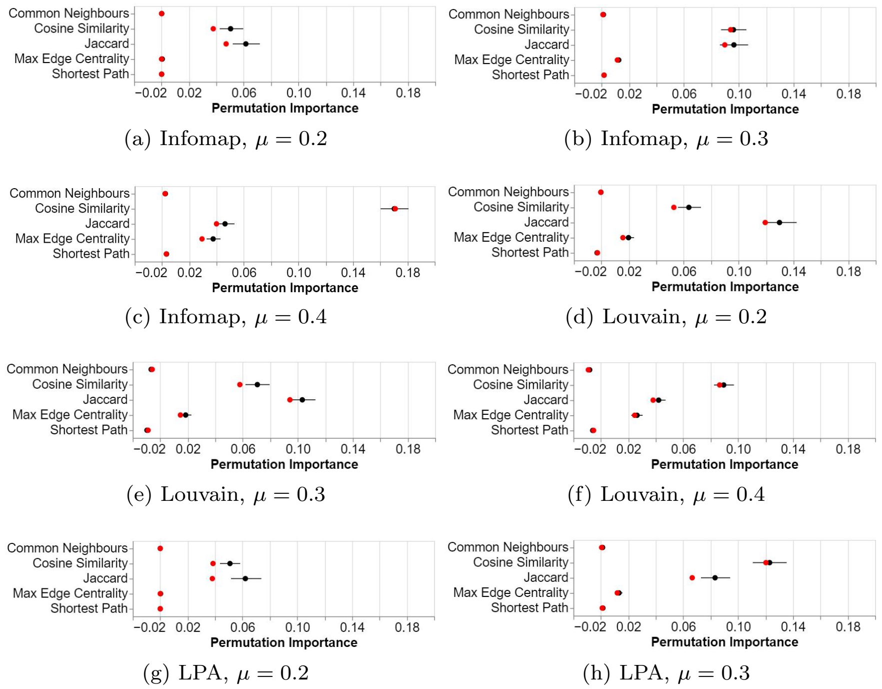

(a) Infomap, $\mu = 0.2$ (b) Infomap, $\mu = 0.3$

(c) Infomap, $\mu = 0.4$ (d) Louvain, $\mu = 0.2$

(e) Louvain, $\mu = 0.3$ (f) Louvain, $\mu = 0.4$

(g) LPA, $\mu = 0.2$ (h) LPA, $\mu = 0.3$

Figure 4: Results of the pair feature experiments

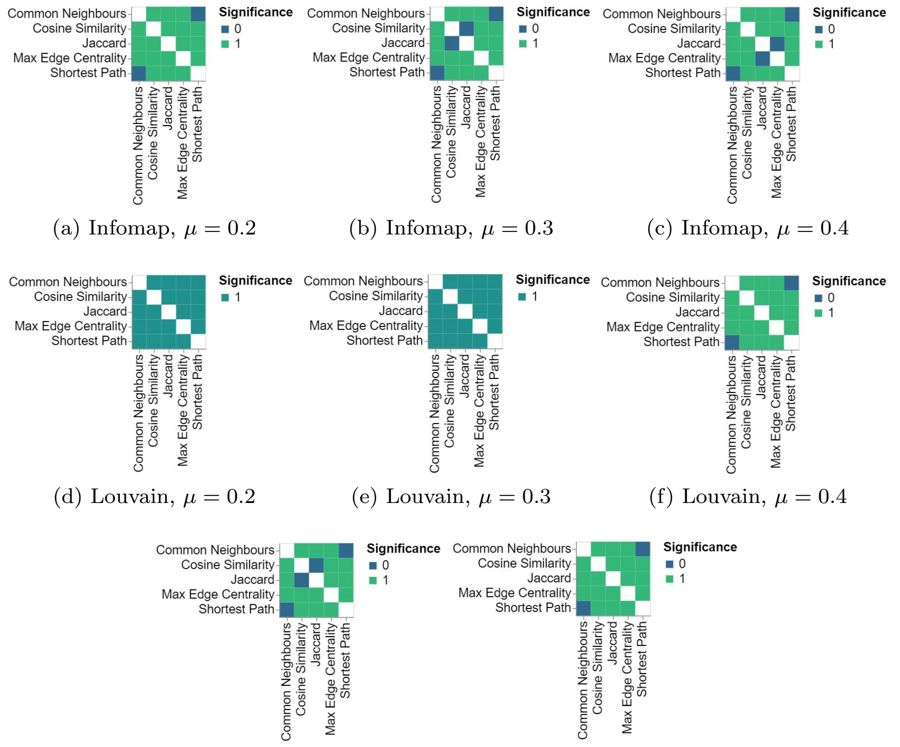

(a) Infomap, $\mu = 0.2$ (b) Infomap, $\mu = 0.3$ (c) Infomap, $\mu = 0.4$

(d) Louvain, $\mu = 0.2$ (e) Louvain, $\mu = 0.3$ (f) Louvain, $\mu = 0.4$

(g) LPA, $\mu = 0.2$ (h) LPA, $\mu = 0.3$

Figure 5: Heatmaps showing significance of Wilcoxon tests on the pair feature experiments

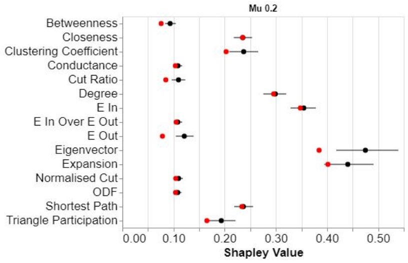

(a) Infomap, $\mu = 0.2$

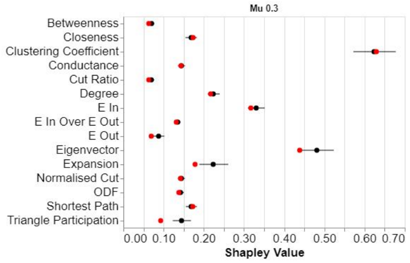

(b) Infomap, $\mu = 0.3$

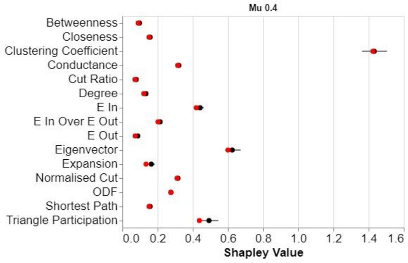

(c) Infomap, $\mu = 0.4$

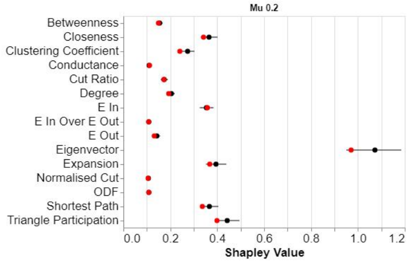

(d) Louvain, $\mu = 0.2$

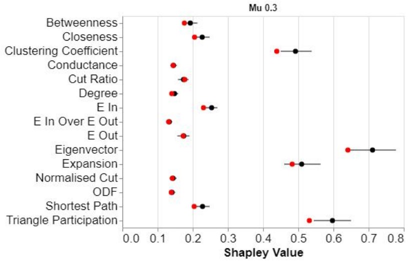

(e) Louvain, $\mu = 0.3$

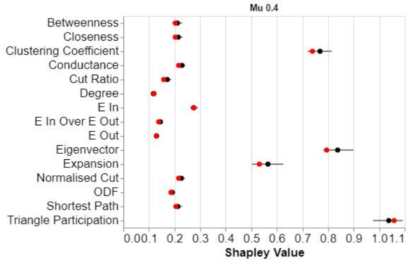

(f) Louvain, $\mu = 0.4$

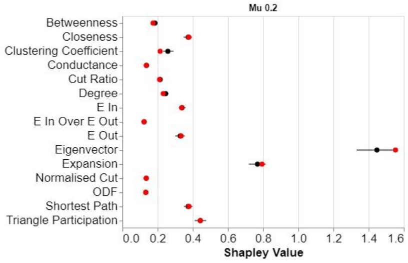

(g) LPA, $\mu = 0.2$

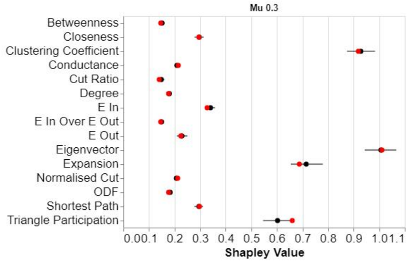

(h) LPA, $\mu = 0.3$

Figure 6: Results of the node feature experiments using Shapley values instead of permutation importance

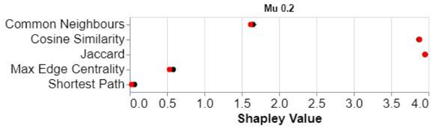

(a) Infomap, $\mu = 0.2$

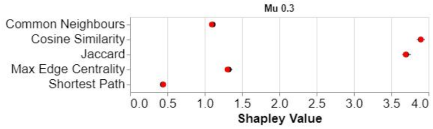

(b) Infomap, $\mu = 0.3$

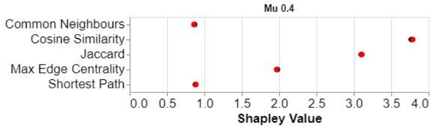

(c) Infomap, $\mu = 0.4$

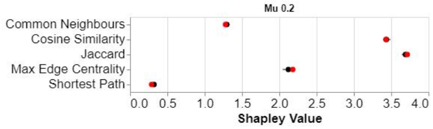

(d) Louvain, $\mu = 0.2$

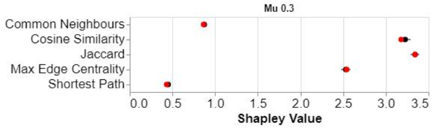

(e) Louvain, $\mu = 0.3$

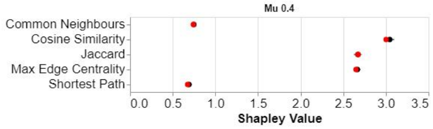

(f) Louvain, $\mu = 0.4$

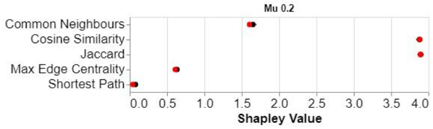

(g) LPA, $\mu = 0.2$

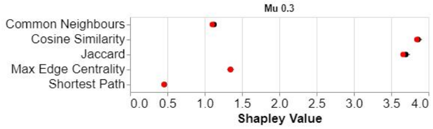

(h) LPA, $\mu = 0.3$

Figure 7: Results of the pair feature experiments using Shapley values instead of permutation importance
